# The Effects of Whole-Body Vibration on the Cross-Transfer of Strength

**DOI:** 10.1100/2012/504837

**Published:** 2012-12-10

**Authors:** Alicia M. Goodwill, Dawson J. Kidgell

**Affiliations:** Centre for Physical Activity and Nutrition Research, School of Exercise and Nutrition Sciences, Deakin University, Melbourne, VIC 3125, Australia

## Abstract

This study investigated whether the use of superimposed whole-body vibration (WBV) during cross-education strength training would optimise strength transfer compared to conventional cross-education strength training. Twenty-one healthy, dominant right leg volunteers (21 ± 3 years) were allocated to a strength training (ST, *m* = 3, *f* = 4), a strength training with WBV (ST + V, *m* = 3, *f* = 4), or a control group (no training, *m* = 3, *f* = 4). Training groups performed 9 sessions over 3 weeks, involving unilateral squats for the right leg, with or without WBV (35 Hz; 2.5 mm amplitude). All groups underwent dynamic single leg maximum strength testing (1RM) and single and paired pulse transcranial magnetic stimulation (TMS) prior to and following training. Strength increased in the trained limb for the ST (41%; ES = 1.14) and ST + V (55%; ES = 1.03) groups, which resulted in a 35% (ES = 0.99) strength transfer to the untrained left leg for the ST group and a 52% (ES = 0.97) strength transfer to the untrained leg for the ST + V group, when compared to the control group. No differences in strength transfer between training groups were observed (*P* = 0.15). For the untrained leg, no differences in the peak height of recruitment curves or SICI were observed between ST and ST + V groups (*P* = 1.00). Strength training with WBV does not appear to modulate the cross-transfer of strength to a greater magnitude when compared to conventional cross-education strength training.

## 1. Introduction

It is well established that unilateral strength training of one limb is capable of eliciting strength gains within the untrained homologous limb [1–5]. As strength transfer commonly occurs in the absence of any changes in muscle hypertrophy, adaptations within the central nervous system (CNS) are likely to modulate the cross-transfer of strength [5, 6]. Recent experimental data has highlighted the role of the primary motor cortex (M1) ipsilateral to the trained limb (iM1) as well as interhemispheric pathways mediating the cross-transfer of strength [4, 6–11].

It is suggested that corticomotor adaptation as well as improvements in strength is largely dependent on the training protocol prescribed [7, 8, 12, 13]. For example, both strength transfer and task-dependant plasticity within the iM1 have been enhanced with high training loads (i.e., greater than 60% 1RM) and when movement speed is controlled via metronome paced training or isokinetic dynamometry [7, 10, 13–17]. Several studies have demonstrated strength increases as well as facilitated corticomotor excitability, reduced short-interval intracortical inhibition (SICI), and silent period duration utilising maximal training loads in both arm and leg muscles [7, 9–11]. Given that the amount of strength gained within the untrained limb is proportional to the strength gained within the trained limb [5, 18], it is desirable to investigate training techniques in which the magnitude of strength transfer can be optimised.

The recent emergence of whole-body vibration (WBV) as a training technique has been of interest to researchers, due to its potential to improve neuromuscular function [19–22]. However, despite the increasing popularity surrounding WBV as a training technique, the evidence for WBV to facilitate strength development to a greater magnitude than conventional strength training alone is inconsistent (for reviews, see [22, 23]). Many studies have reported increases in strength following an acute bout of WBV [24–26]. Similarly, increases in strength have also been demonstrated following a period of strength training with the addition of WBV [27–34], suggesting that WBV training may be an effective and alternative training technique for strength development [23]. However, more recent studies have shown that a range of strength training protocols (including low, moderate, and heavy training loads) with superimposed WBV have no additional benefit on strength development when compared to conventional strength training [35–42]. The inconsistencies amongst the studies above are most likely related to variations in training protocols, training modes (in particular bilateral lower limb training), and participant training status as well as differences in vibration application (i.e., vertical versus rotational) and parameters (i.e., frequency and amplitude). One further consideration is that the aforementioned studies prescribed exposure to WBV during training of both limbs; however, it is not known as to whether WBV, combined with cross-education strength training, can improve strength of the opposite untrained limb.

Although increases in strength have been observed following WBV [25, 30, 32, 34], the neural mechanisms underpinning these changes remain unclear. Suggested mechanisms for improved neuromuscular function have been derived from responses to local muscle vibration. These include increased corticomotor excitability and decreased short-interval intracortical inhibition (SICI) [43], increased muscle activity due to dampening of the vibrational oscillations [44–46], increased motor unit activity [47–50], and the tonic vibration reflex [51]. Although previously debated as to whether local and WBV share similar mechanisms, the few studies examining physiological responses during WBV demonstrate some similarities. For example, Pollock et al. [48] demonstrated that the motor unit firing patterns were phase locked during WBV, representing stimulation of monosynaptic pathways (1a afferents). This evidence suggests that mechanisms associated with the tonic vibration reflex may be present to a some degree during WBV [48, 52]. Additionally, Mileva et al. [53] suggested enhanced excitability of the corticomotor pathway during WBV as well as modulation of intracortical circuits [53]. Based upon these recent findings regarding the potential neural mechanisms associated with WBV, it is possible that repeated bouts of cross-education strength training in combination with WBV may modulate corticomotor plasticity to a greater extent compared to conventional cross-education strength training alone; however this currently has not been examined. Currently, no study has utilised paired pulse TMS to investigate the effects of cross-education strength training with the addition of WBV on corticomotor excitability and SICI within the iM1, which may mediate the cross-transfer of strength. As strength transfer is proportional to the amount of strength gained, investigating techniques which enhance cross-education are clinically important for populations with reduced capacity to train or use one limb, such as limb immobilisation following surgery [54]. Therefore, it was of interest to the current study to examine whether the addition of WBV would enhance the cross-transfer of strength. It was hypothesised that WBV would modulate corticomotor excitability and SICI within the iM1, leading to an increase in strength transfer to the untrained limb compared to conventional cross-education strength training.

## 2. Methods

### 2.1. Experimental Design

This study consisted of an interparticipant repeated measure design, whereby individuals were randomly allocated to a strength training (ST), a strength training with WBV (ST + V), or a control group. One week prior to the intervention, participants undertook a familiarisation session involving learning the correct exercise technique, exposure to WBV, and exposure to all testing procedures, to minimise the effect of learning. Both the ST and ST + V groups completed 9 supervised cross-education strength training sessions over a 3-week period. Testing measures included unilateral squat single repetition maximum (1RM) strength and maximal voluntary isometric contraction (MVIC) torque (trained and untrained legs), muscle thickness via imaging ultrasound, corticomotor excitability (recruitment curves), and SICI via single and paired pulse TMS. All testing visits lasted approximately 60 minutes, and all training sessions were fully supervised and took approximately 20 minutes.

### 2.2. Participants

Twenty-one healthy individuals aged between 18 and 35 years (*m* = 9, *f* = 12) were recruited from the university population. All participants provided written informed consent prior to participation. Following participant information questionnaires, only dominant right leg [55] individuals as well as untrained individuals that had not partaken in lower body strength training within the past 6 months were included in the sample. Participants were randomly (according to baseline strength and gender) allocated to a ST (*n* = 7, 21 ± 1.1 years), ST + V (*n* = 7, 22 ± 2.1 years), or a control group (*n* = 7, 21 ± 1.2 years).

The number of participants required was based on power calculations for the expected changes in mean rectified MEPs (sEMG recordings from the rectus femoris muscle). Using previous cross-education data in healthy untrained adults [10], we estimated that 6 participants in each group would provide at least 80% power (95% confidence interval) to detect a 15% difference in mean rectified MEPs assuming a SD of 10–15% between groups at *P* < 0.05 (two tailed).

### 2.3. Maximum Strength Testing

Maximum voluntary dynamic strength of all participants was determined by a 1RM single leg squat. All participants completed a warmup that consisted of 5-minute moderate aerobic exercise on a cycle ergometer and 2 warmup sets of single leg squats with increasing weight. The 1RM test involved performing single leg squats positioned under a power rack (Nautilus XPLOAD, VA, USA). Squat depth was determined by using an electromagnetic goniometer (3DM-GX2, Williston, VT, USA) to control for knee joint angle (80°). The starting weight was determined by the participants estimate of his/her leg strength. If the estimated weight was successful, the weight was then increased until the participant could no longer perform 1 repetition. The last successful trial was recorded as their 1RM strength. Between each trial, a 3-minute rest period was allocated to minimise muscular fatigue. This procedure was performed for both legs. Additionally, isometric torque was determined using an isokinetic dynamometer (Biodex system 4 Pro, Biodex Medical Systems, Shirley, IN, USA) prior to and following the training intervention, to control for background muscle activity during TMS testing. Participants were placed in a seated position with a trunk-thigh angle of 110°. The axis of the dynamometer was then aligned with the anatomical axis of the knee joint, and the leg was held to the dynamometer lever arm using a padded strap, positioned 1 cm superior to the malleoli of the ankle. In order to ensure that the trunk was stabilised during testing, a waist strap and two cross-over shoulder straps were used. During isometric testing, the knee was positioned at a 60° angle and the participant was required to perform 3 maximal isometric leg extensions for 5 seconds with a 5-second rest period between each repetition. The highest peak torque of the 3 trials was taken and recorded as the participant MVIC torque.

### 2.4. Measurement of Anterior Thigh Muscle Thickness

Muscle thickness of both the trained and untrained anterior thigh was measured on a SonoSite Ultrasound (Springfield, NJ, USA), to quantify changes in muscle hypertrophy. The site of measurement was determined by placing the transducer perpendicular to the long axis of the thigh on its superior aspect, three-fifths from the ASIS to the superior patella border [56]. A 6–15 Hz transducer probe was lubricated with transmission gel and placed lightly on the marked area. The image was obtained while the participants laid supine with their legs hip width apart and knees straight. When a clear image was visible on the monitor, the pressure of the transducer was slowly reduced to ensure minimal compression of the muscle before the image on the monitor was frozen. To ensure accuracy of the data before and after testing, marking sites were recorded and matched at each testing session. Reliability for ultrasound testing was demonstrated prior to data collection with a coefficient of variance (CoV) of less than 1% for the left (*P* = 0.11; *r* = 0.99) and right (*P* = 0.64; *r* = 0.99) legs.

### 2.5. Transcranial Magnetic Stimulation and Surface Electromyography

TMS was applied over the cortical representation of the quadriceps muscle group, using a circular coil (90 mm diameter) attached via a BiStim unit, to 2 Magstim 200^2^ stimulators (Magstim, Dyfed, UK) [57]. MEPs were produced by stimulation of the contralateral M1, innervating the untrained left leg during low level (10% MVIC) background muscle activity. The handling of the TMS coil was positioned over the vertex of the head and held tangential to the skull in an anterior-posterior orientation, so the current flowed in a counterclockwise direction for activating the rectus femoris of the untrained left leg. To ensure consistency during and between testing sessions, all participants were fitted with a semitransparent cap in relation to the nasion-inion and interaural lines. The cap was marked with points 1 cm apart in a longitude-latitude matrix, to allow the site evoking the largest MEP in the rectus femoris muscle (i.e., optimal site) to be explored, marked, and recorded. Active motor threshold (AMT) was determined by the lowest stimulus required to produce an MEP with peak-peak amplitude of at least 200 *μ*V in 3 out of 5 trials, during low-level voluntary knee extension.

sEMG was recorded from the left rectus femoris muscle using bipolar Ag-AgCl electrodes. These electrodes were placed on the rectus femoris, three-fifths of the distance between the ASIS and the upper border of the patella, with an interelectrode distance (centre to centre) of 20 mm. The reference electrode was placed on the patella to ensure that no muscle activity was recorded. All cables were fastened with tape to prevent movement artefact. The area of electrode placement was shaven to remove fine hair, rubbed with an abrasive rasp to remove dead skin, and then cleaned with 70% isopropyl alcohol. The exact sites were marked with a permanent marker by tracing around the electrode, and this was maintained for the entire 3-week period by both the researcher and participant to ensure consistency of electrode placement relative to the innervation zone. An impedance meter was used to ensure that impedance did not exceed 10 kΩ prior to testing. sEMG signals were amplified (×1000) with bandpass filtering between 20 Hz and 1 kHz and digitised at 2 kHz for 500 ms, recorded, and analysed using a PowerLab 8/35 (ADInstruments, Australia).

### 2.6. Recruitment Curves

Once AMT was established, the stimulus intensities required to create the TMS recruitment curve were determined. Stimulus intensities began at 10% of maximum stimulator output (MSO) below AMT and increased in 5% of MSO increments up to 40% of MSO above AMT to ensure a plateau in MEP amplitude. A single block consisted of 15 stimuli at a single intensity (approximately 6–9 sec separating each stimulus), and the order of presentation of the blocks was randomised throughout the trial according to a predetermined randomisation protocol.

### 2.7. Short-Interval Intracortical Inhibition

The protocol for SICI included 15 unconditioned (single pulse at 1.2 × AMT) test stimuli and 15 conditioned stimuli to induce SICI. The pair of stimuli to induce SICI consisted of a subthreshold (0.7 × AMT) conditioning stimulus followed by a suprathreshold (1.2 × AMT) test stimulus, with an ISI of 3 ms [58]. Single and paired pulse stimuli were presented according to a predetermined randomisation protocol, with 6–9 seconds between each stimulus.

### 2.8. M-Waves

Direct muscle responses were obtained under resting conditions from the left rectus femoris by supramaximal percutaneous electrical stimulation of the femoral nerve, approximately 3–5 cm below the inguinal ligament in the femoral triangle. A digitimer (Hertfordshire, UK) DS7A constant-current electrical stimulator (pulse duration 1 ms) was used to deliver each electrical pulse. An increase in current strength was applied to the femoral nerve until there was no further increase in the amplitude of sEMG response (M_MAX_). To ensure maximal responses, the current was increased by an additional 20% and the average M_MAX_ was obtained from 5 stimuli, with 6–9 seconds separating each stimulus.

### 2.9. Training Protocol

Participants in the ST and ST + V groups undertook supervised unilateral strength training of their dominant leg, 3 times per week for 3 weeks. Both groups underwent identical protocols with the only difference being the addition of WBV. The strength training program prescribed was progressively overloaded and periodised based on their maximum single leg squat strength of their dominant leg in the pretesting session and then adjusted as necessary for the 3 week intervention. Prior to each session, a 5-minute warmup was performed on a cycle ergometer at an intensity of 70% age-predicted maximum heart rate (±5%) to increase muscle temperature and blood flow. This was followed by 1 set of single leg squats at 12RM and 1 set at 10RM. Participants then completed their prescribed training. Participants completed a prescribed training load of 4 sets at 75% of their 1RM (8 repetitions) in week 1, 77.5% of their 1RM (8 repetitions) in week 2, and 80% of their 1RM (8 repetitions) for week 3. Repetition timing was set at 3 seconds for the concentric phase and 4 seconds for the eccentric phase via the use of an electronic metronome. sEMG electrodes were placed on the rectus femoris muscle of the contralateral leg, which remained relaxed behind the participant resting on a 20 cm box, and visual feedback of muscle activation was provided to the participant and investigator via an oscilloscope (HAMEG, Mainhausen, Germany) that was located 1 m in front of them at eye level, to minimise muscle activation of the rested leg during training.

Both groups performed all training on the vibration platform; however, for the ST group the machine was switched off. Participants in the ST + V group were exposed to vertical sinusoidal vibration (Power Plate Next Generation, Northbrook, IL, USA), placed under the power rack in a conventional starting squat position, with the untrained leg resting on a 20 cm box behind them. In accordance with previous literature, the vibration frequency was set and validated at 35 Hz [34] and the peak-to-peak displacement (displacement = 2.5 mm, acceleration = 32.08 m·s^−1^) was recorded from a multiple axis Nanotrack (Catapult, Melbourne, VIC, Australia) fixed to the vibration platform at the marked foot position. From this position, exposure to vibration was equal to the time taken to complete 8 repetitions at 3 seconds concentric and 4 seconds eccentric repetition timing (i.e., 56 seconds). The appropriate foot position was marked on the vibration platform to ensure consistency between training sessions [59].

### 2.10. Data Analyses

Procedures outlined by Kidgell et al. [7] were applied to quantify the contralateral transfer of strength following the 3-week intervention. The difference in change in mean strength of the untrained left leg in the experimental groups and the control group post intervention was used to determine the strength transfer percentage. The calculation was performed as follows:

(1) $$\begin{array}{c} \left(\frac{E_{\text{Post}}-E_{\text{Pre}}}{E_{\text{Pre}}}-\frac{C_{\text{Post}}-C_{\text{Pre}}}{C_{\text{Pre}}}\right)100, \end{array}$$

where

- *E*_Post_ is the mean posttraining 1RM for the strength or WBV groups untrained leg,
- *E*_Pre_ is the mean pretraining 1RM for the strength or WBV groups untrained leg,
- *C*_Post_ is the mean posttraining 1RM for the control groups untrained leg,
- *C*_Pre_ is the mean pretraining 1RM for the control groups untrained leg.

Prestimulus root mean square (rms) EMG (*μ*V) was determined in the rectus femoris over a 20 ms period prior to each TMS stimulus before and after testing. rmsEMG was also recorded from the left untrained rectus femoris during training, to minimise any potential mirror activity within the untrained left leg. MEP amplitudes were analysed using PowerLab (ADInstruments, Australia) software after each stimulus was automatically flagged with a cursor, providing peak-to-peak values in *μ*V, and were then normalised to M_MAX_. Recruitment curves were constructed by plotting stimulus intensity against MEP amplitude (% of M_MAX_). The slope, peak height (plateau) values, and the stimulus intensity at which MEP amplitude is halfway between top and bottom (*V*_50_) were determined by applying a nonlinear Boltzmann sigmoidal equation using Prism5 (GraphPad Software Inc., CA, USA):

(2) $$\begin{array}{c} \mathrm{MEP}\left(s\right)=\text{Bottom}+\frac{\left(\text{Top}-\text{Bottom}\right)}{1+\exp\left(\left(V_{50}-X\right)/\text{Slope}\right)}, \end{array}$$

where

- *s* represents stimulus intensity,
- Top represents the *MEP*⁡ plateau value (peak height),
- Bottom represents the minimum *MEP*⁡ values,
- *V*_50_ represents the stimulus intensity at which *MEP*⁡ amplitude is halfway between top and bottom,
- Slope represents the steepness of the curve.

SICI was quantified by dividing the average paired pulse *MEP*⁡ by the average single pulse *MEP*⁡ at 1.2 × AMT and multiplying by 100.

### 2.11. Statistical Analyses

All data was screened for normal distribution using the Shapiro-Wilks test, with the data being judged as normally distributed (*P* > 0.05). Consequently, the following parametric analyses were performed. A two (time) by three (condition), repeated measure analysis of variance (ANOVA) was used to determine the effects of strength training with WBV on all dependant variables (strength, recruitment curves, SICI, and muscle thickness). Where appropriate, pairwise post hoc comparisons with Bonferroni correction (*P* < 0.016) were employed. An additional two (condition) by three (time) two-way repeated measure ANOVA was conducted in order to determine whether any differences in muscle activation occurred in the untrained leg, within and between groups across the 3-week intervention. Intraclass correlation coefficients (ICCs), CoV, and paired *t*-tests were used to determine the reliability of the ultrasound testing protocol. Alpha was set at *P* < 0.05.

## 3. Results

### 3.1. Muscle Thickness

There were no differences in muscle thickness of the trained right leg between the groups at baseline (*F*_2,18_ = 1.87; *P* = 0.19). There was a main effect for time (*F*_1,18_ = 9.49; *P* = 0.007); however, no main effect for group (*F*_2,18_ = 0.47; *P* = 0.63) or group by time interactions (*F*_2,18_ = 1.36; *P* = 0.28) was detected following training. Similarly, muscle thickness did not differ significantly in the left leg between the groups at baseline (*F*_2,18_ = 1.50; *P* = 0.25). There were no main effects for time (*F*_1,18_ = 2.65; *P* = 0.12), group (*F*_2,18_ = 0.54; *P* = 0.59), or group by time interactions following the intervention (*F*_1,18_ = 1.38; *P* = 0.28).

### 3.2. Voluntary Dynamic Strength (1RM)

**Figure 1:**
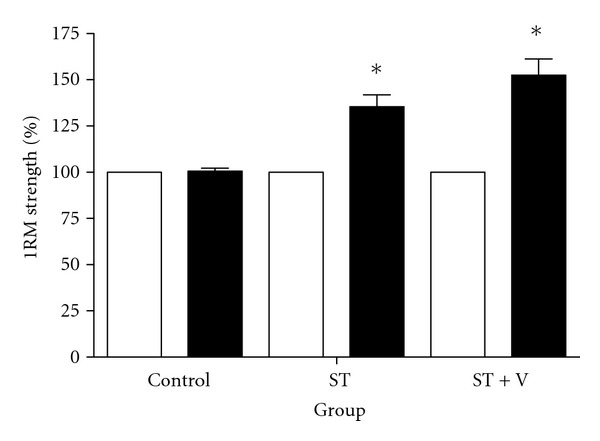
Mean ± SE 1RM strength (expressed as a percentage change) for all groups before (light bars) and after training (dark bars). *denotes an increase in strength following training (*P* < 0.016). There were no differences in strength between the ST and ST + V groups following training (*P* = 0.15).

For the trained right leg, there was no difference in 1RM strength between the groups at baseline (*F*_2,18_ = 4.94; *P* = 0.20). Following training, there was a main effect for time (*F*_1,18_ = 74.82; *P* < 0.001), group (*F*_2,18_ = 17.90; *P* < 0.001), and group by time interaction (*F*_2,18_ = 17.90; *P* < 0.001). Post hoc analyses demonstrated increases in strength in both the ST (40.67%; ES = 1.39; *P* = 0.002) and ST + V (55.05%; ES = 1.03; *P* < 0.001) groups compared to control; however, there were no differences in the magnitude of strength gain between the ST and ST + V groups (*P* = 0.32). Similarly for the left untrained leg, groups did not differ in 1RM strength at baseline (*F*_2,18_ = 2.75; *P* = 0.09). Following training, there was a main effect for time (*F*_1,18_ = 81.58; *P* < 0.001) and group (*F*_2,18_ = 21.24; *P* < 0.001) as well as a group by time interaction (*F*_2,18_ = 21.24; *P* < 0.001). Post hoc analyses revealed a 1RM strength increased in both the ST (35.40%; ES = 0.99; *P* = 0.001) and ST + V (52.55%; ES = 0.98; *P* < 0.001) groups compared to control (Figure 1); however, no difference in strength was observed between ST and ST + V (*P* = 0.15).

**Figure 2:**
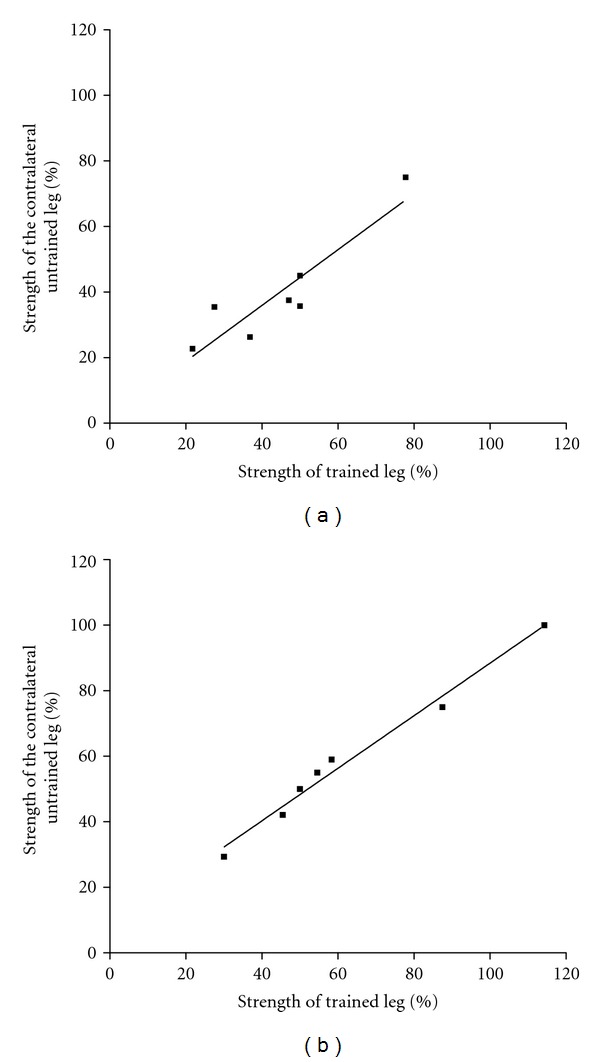
Mean strength (expressed as a percentage change) of the trained right and untrained left leg post training, for ST (a) and ST + V (b) groups.

There was a positive correlation between the percentage of strength gained in the trained right leg and the percentage of strength transfer to the contralateral untrained left leg for both the ST (*r* ^2^ = 0.83; *P* = 0.004; Figure 2(a)) and ST + V group (*r* ^2^ = 0.98; *P* < 0.001; Figure 2(b)). Cross-education strength training of the right leg resulted in a 35.40% and 52.09% strength transfer to the contralateral untrained left leg, for both ST and ST + V groups, respectively.

### 3.3. rmsEMG

At week one, there were no differences in rmsEMG in the untrained leg during training between the participants (*F*_1,24_ = 2.59; *P* = 0.13). Over the 3-week intervention, there was no main effect for time (*F*_2,24_ = 0.10; *P* = 0.90), group (*F*_1,24_ = 0.79; *P* = 0.39), or group by time interactions (*F*_2,24_ = 1.02; *P* = 0.37).

In addition, for the untrained left leg of all groups, rmsEMG (*μ*V) 20 ms prior to TMS stimulation at 10% MVIC before and after testing, revealed no main effects for time (*F*_1,18_ = 0.44; *P* = 0.51), group (*F*_2,18_ = 0.44; *P* = 0.65), or group by time interactions (*F*_2,18_ = 0.31; *P* = 0.74).

### 3.4. Active Motor Threshold and Motor Evoked Potentials

For the untrained left leg, no differences in stimulator output at AMT were present between groups at baseline (*F*_2,18_ = 3.17; *P* = 0.07). There was a main effect for time (*F*_1,18_ = 5.51; *P* = 0.03); however, no main effect for group (*F*_2,18_ = 0.11; *P* = 0.89) or group by time interactions was detected following the intervention (*F*_2,18_ = 1.64; *P* = 0.22).

### 3.5. Recruitment Curves

**Figure 3:**
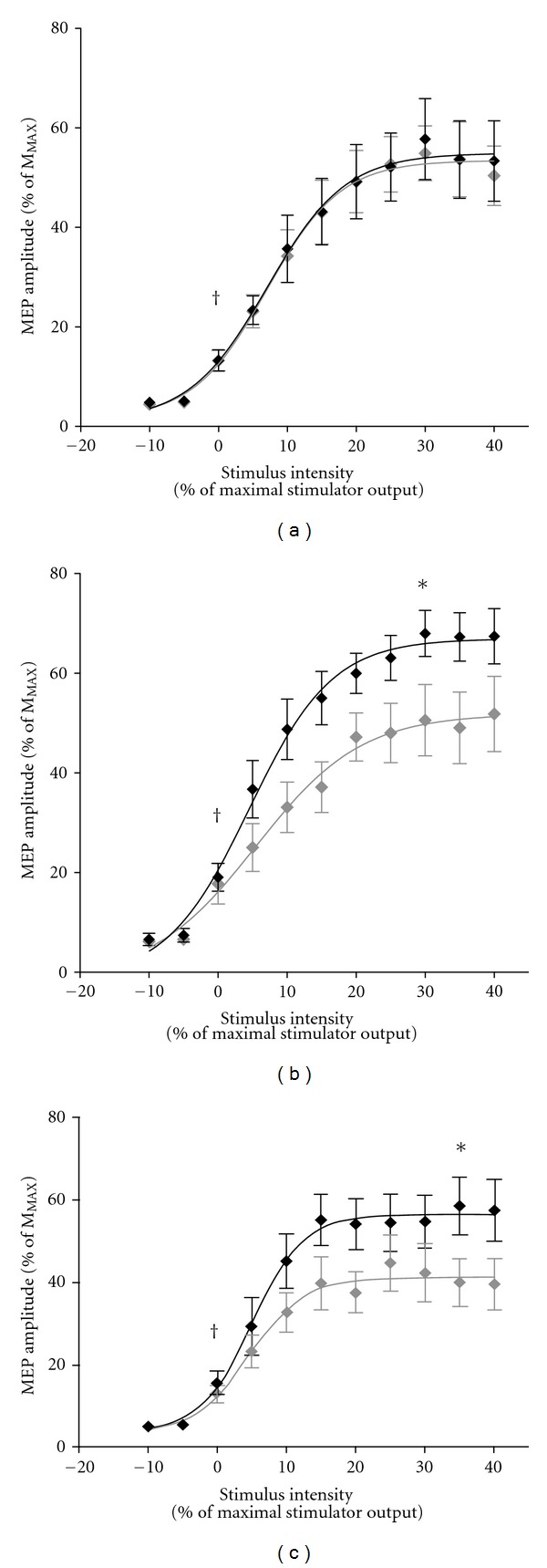
Mean ± SE MEP amplitudes (expressed as a percentage of M_MAX_) obtained from the left untrained rectus femoris for the control (a), ST (b), and ST + V (c) groups before (light curve) and after (dark curve) training. Each recruitment curve is characterised by AMT, estimated slope and peak height (plateau), and the stimulus intensity at which the MEP amplitude is 50% of the maximum MEP (*V*_50_). ^†^identifies AMT. *denotes significant increases in peak height post training (*P* < 0.016). There were no differences in peak height between the ST and ST + V groups following training (*P* = 1.00).

Recruitment curves were constructed to determine properties including the slope, peak height, and half-peak slope (*V*_50_) prior to and following the training intervention. There were no main effects for time, group, or group by time interactions for the slope *V*_50_ following training (*P* > 0.05). For the untrained left leg, no differences in peak height of the recruitment curves were observed at baseline (*F*_2,18_ = 0.26; *P* = 0.77). There was a main effect for time (*F*_1,18_ = 31.36; *P* < 0.001) and group (*F*_2,18_ = 8.40; *P* = 0.004) as well as a group by time interaction (*F*_2,18_ = 8.40; *P* = 0.004). Post hoc revealed a 32% increase in peak height for the ST group (*P* = 0.11; Figure 3(b)) and a 34% increase for the ST + V group (*P* = 0.10; Figure 3(c)) compared to control (Figure 3(a)); however, no differences between ST and ST + V groups were detected (*P* = 1.00).

### 3.6. Short-Interval Intracortical Inhibition

**Figure 4:**
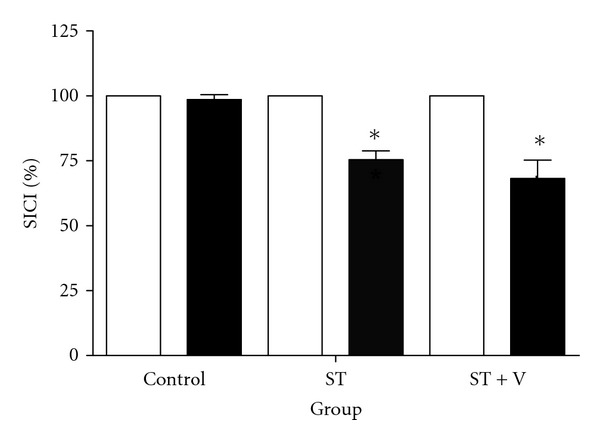
Mean ± SE SICI (expressed as a percentage change) for all groups before (light bars) and after training (dark bars). *denotes a significant reduction in SICI following training for the ST and ST + V groups (*P* < 0.016); however, no differences in SICI following training were observed between the ST and ST + V groups (*P* = 1.00).

There were no differences in SICI between the groups for the left leg at baseline (*F*_2,18_ = 0.59; *P* = 0.57). There was a main effect for time (*F*_1,18_ = 48.73; *P* < 0.001), group (*F*_2,18_ = 11.29; *P* = 0.001), and group by time interaction (*F*_2,18_ = 11.29; *P* = 0.001). Post hoc revealed that SICI was reduced by 24.56% for ST (*P* = 0.001) and 31.84% for the ST + V (*P* = 0.006) group compared to control (Figure 4), with no differences observed between the ST and ST + V groups (*P* = 1.00).

## 4. Discussion

Cross-education strength training resulted in increased strength in the untrained limb, accompanied by facilitated corticomotor excitability and a reduction in SICI within the iM1. The most important finding was that the addition of WBV to cross-education strength training did not confer any advantage on strength transfer, corticomotor excitability, or SICI greater than conventional cross-education training.

### 4.1. Dynamic Voluntary Strength (1RM)

Cross-education training resulted in a 35% and 52% strength transfer to the untrained leg in both the ST and ST + V groups, respectively. Interestingly, although the percentage of transfer was 15% greater in the ST + V group, this did not reach statistical significance. Effect size analysis (0.99 for ST and 0.98 for ST + V) showed no differences in strength transfer between the two training groups. These findings are consistent with recent investigations reporting no differences in strength between bilateral squat training with or without WBV, in both healthy adults and athletes [35, 36, 38, 40]. The concept of external loading (i.e., body mass + barbell weight) is an important factor when considering the benefits of WBV combined with strength training. Although the vibration parameters were validated in this study, evidence suggests that the addition of heavy external loads to the vibration plate may alter the true acceleration of oscillations imparted upon the neuromuscular system [60]. As the present study only prescribed a unilateral training load, we expected that oscillations from WBV would still be effective. However, the nonsignificant differences in strength transfer between the training groups in the present study imply that increased muscle activity and stiffness induced by the external load may have acted to dampen the vibratory oscillations imparted on the soft tissue structures [46]. Based on the current findings, as well as previous studies employing both light and heavy external loads [35, 36], it is likely that the addition of WBV to cross-education strength training may be counterproductive.

Although WBV did not produce an additive effect on the cross-transfer of strength, the magnitude of strength transfer observed in both training groups was significantly higher than previous cross-education studies [4, 7, 9, 61, 62]. These differences may be due, in part, to other training techniques prescribed in the current study, rather than the addition of WBV itself. For example, Shima et al. [62] observed an 8.9% increase in isometric strength after 6 weeks of isotonic training, highlighting the importance of specificity between training and testing to produce accurate maximal strength changes. Our findings are comparable to recent cross-education studies by Kidgell et al. [7] and Latella et al. [11] who observed a 19.2% and 17.4% strength transfer, following 4 weeks of unilateral bicep curl and 8 weeks of leg press training, respectively. Interestingly, the strength transfer is also comparable to that observed by Hortobágyi et al. [13] who observed a 77% increase in strength following eccentric contractions. An aspect to the present study that may have contributed to a slightly larger strength transfer may have been the complexity of the training task, particularly in novice individuals. Even though familiarisation was conducted to reduce learning, the complexity and skill required to perform a unilateral squat, timed to an externally paced metronome, may have contributed to the acquisition of strength [16, 63, 64].

### 4.2. Corticomotor Plasticity

There is little evidence examining the effect of WBV on corticomotor excitability and SICI during and following WBV [36, 53]. In the present study, similar to strength transfer, the addition of WBV did not increase corticomotor excitability in the iM1 any greater when compared to conventional cross-education strength training. Nevertheless, 3 weeks of unilateral training resulted in increased corticomotor excitably for both training groups, as evident by increased amplitude of MEPs and peak height of the recruitment curve. Changes in the properties of recruitment curves represent adjustments in synaptic efficacy, possibly through strengthening of existing corticomotor projections [65]. These findings are in agreement with previous cross-education data showing augmented MEPs within the iM1 following strong unilateral contractions [7, 66–68]. Therefore, the present data reinforces the role of the iM1 underpinning the cross-transfer of strength.

The novelty of the current study was a reduction in SICI in the iM1 in both training groups. Consistent with the other variables (strength and corticomotor excitability), the magnitude of SICI did not differ between the two training groups. Although this is the first study to assess the effect of WBV training on SICI modulating the cross-transfer of strength, the present data is comparable to the few bilateral WBV studies utilising paired pulse TMS. Our findings are in contrast to those by Mileva et al. [53], who observed an increase in SICI *during* WBV, but are consistent with Weier and Kidgell [36], showing that the addition of WBV did not modulate SICI greater than conventional strength training. Our findings suggest that there may be different physiological responses occurring *during* WBV and *following* a period of strength training with WBV. Given that there were no differences in SICI in the iM1 between the training groups, it is likely that the complexity and skilled nature of the training protocol itself facilitated the reduction in SICI. This has recently been supported by studies showing that strictly controlled motor paced training results in use-dependent plasticity within intracortical circuits [16, 69, 70]. Certainly, studies have reported a reduction in SICI in the iM1 following complex unimanual motor skill training when compared to a simple motor task [68–70]. This data supports the concept that task acquisition occurs, in part, due to changes in GABA-mediated SICI [71]. Additionally, it has been demonstrated that SICI is reduced to a greater magnitude with increasing force production [68, 72]; therefore, the training load prescribed in the current study may have also been a contributing factor to the reduction in SICI within the iM1.

The training-related reductions in intracortical inhibition are likely to be influenced by changes in the strength of corticomotor connections [73], possibly contributing to the increased excitability within the iM1 and increased voluntary drive to the untrained limb observed in this study. It is known that activation of both agonist and synergistic muscles occurs during voluntary contractions [74, 75]. Moreover, there is good evidence to suggest that SICI is reduced prior to and during the activation of both agonists as well as synergistic muscles [72, 76, 77]. Although the present study only recorded SICI from one muscle, a reduction in SICI from trained synergistic muscles may have also contributed to increased voluntary drive to the untrained limb.

Recently, cross-education data has suggested that reduced SICI within the iM1 may be attributed to reductions in interhemispheric inhibition as a result of repeated voluntary contractions, occurring through transcallosal pathways [9]. In line with the findings from a previous cross-education study [10], it appears that reduced SICI within the iM1, possibly as a result of reduced interhemispheric inhibition, is an important factor modulating motor output to the untrained limb.

### 4.3. Limitations and Future Research

The present findings show that the addition of WBV does not appreciably modulate the cross-transfer of strength; however, we should consider some potential limitations. Although the vibration parameters were validated and remained consistent between training groups, the gravitation load (i.e., the participants body mass and weight of the barbell) may have varied the accelerations imparted upon each individual. Therefore, individualised frequencies and amplitudes may be needed to provide a true representation of the neuromuscular effects of WBV. Further studies are also required to determine the optimal gravitational training load for WBV to have an advantageous effect on strength development. As we did not measure corticomotor adaptations contralateral to the trained limb, it cannot be certain whether the same adaptations facilitated the strength gains observed in the exercised limb. Despite this, mechanisms mediating cross-education in both the contralateral and ipsilateral M1 have been previously established [5, 7, 9, 10] and support the concept that improved motor output is partially attributed to contralateral cortical activation. Finally, although cross-education and WBV are thought to have little effect on spinal reflexes [78–81], this was not quantified in the present study; therefore, the role of the spinal cord mediating the cross-transfer of strength, following WBV training, cannot be ruled out.

## 5. Conclusions

In conclusion, the present data is the first to demonstrate that WBV does not appear to modulate the cross-transfer of strength or underlying corticomotor plasticity to a greater extent compared to conventional cross-education strength training. Our findings show that the prescription of training variables, rather than the addition of WBV, is fundamentally important in modulating corticomotor adaptations underpinning the cross-transfer of strength. The present findings have important implications towards the prescription of cross-education strength training as a potential rehabilitation method to preserve or develop strength in patient populations that may have limited movement or are unable to use one limb.

## Abbreviations

1RM: Single repetition maximum
AMT: Active motor threshold
iM1: Primary motor cortex ipsilateral to the trained limb
M1: Primary motor cortex
MSO: Maximal stimulator output
MEP: Motor evoked potential
M_MAX_: Maximum compound wave (M-wave)
rmsEMG: Root mean square electromyography
sEMG: Surface electromyography
SE: Standard error
SICI: Short-interval intracortical inhibition
ST: Strength training
ST+V: Strength training with whole-body vibration
TMS: Transcranial magnetic stimulation
WBV: Whole-body vibration.
